# Comparative Effects of Low- and High-Volume HIIT Versus Yoga on Psychological Health and Physical Fitness in Female College Students with Binge Eating: An 8-Week Three-Arm Randomized Controlled Trial

**DOI:** 10.3390/nu18132180

**Published:** 2026-07-04

**Authors:** Chen Tian, Manli Lin, Yizhen Yan, Yiting Li, Lu Guo, Li Zhao, Shanshan Mao

**Affiliations:** 1 Beijing Key Laboratory of Sports Performance and Skill Assessment, Beijing Sport University, Beijing 100084, China; 2024210410@bsu.edu.cn; 2 School of Sports Medicine and Rehabilitation, Beijing Sport University, Beijing 100084, China; bsu_linmanli@outlook.com (M.L.); yanyiz@126.com (Y.Y.); 1791056@bsu.edu.cn (Y.L.); 3 Key Laboratory for Performance Training and Recovery of General Administration of Sport, Beijing Sport University, Beijing 100084, China; 4 School of Psychology, Beijing Sport University, Beijing 100084, China; guol@bsu.edu.cn

**Keywords:** low-volume high-intensity interval training, high-volume high-intensity interval training, yoga, binge eating, negative emotional states, physical fitness

## Abstract

**Background**: Binge eating (BE) is frequently associated with negative emotional states, obesity, and physical inactivity. Although yoga may improve binge eating and emotional symptoms, its effects on physical fitness remain unclear. In contrast, high-intensity interval training (HIIT) has been demonstrated to effectively enhance physical fitness. This study compared the effects of low-volume HIIT (LV-HIIT), high-volume HIIT (HV-HIIT), and yoga on binge eating, negative emotional states, and physical fitness in female college students with binge eating. **Methods**: Fifty-five physically inactive female college students with binge eating (BES ≥ 18) were randomly assigned to LV-HIIT (*n* = 19), HV-HIIT (*n* = 18), or yoga (*n* = 18) for 8 weeks. The Binge Eating Scale (BES), Depression Anxiety Stress Scale-21 (DASS-21), body fat percentage, waist circumference, and maximal oxygen uptake (VO2max) were assessed before and after the intervention. Data were analyzed using intention-to-treat linear mixed models, with per-protocol repeated-measures ANOVA as a supplementary analysis. **Results**: After 8 weeks of intervention, significant improvements over time were observed across all groups in binge eating, negative emotional states, and cardiorespiratory fitness (all *p* < 0.05). Waist circumference and body fat percentage did not change significantly in the ITT analysis. No significant time × group interaction effects were detected for any outcome (all *p* > 0.05), indicating that the improvements did not differ significantly among the LV-HIIT, HV-HIIT, and yoga groups. **Conclusions**: An 8-week intervention of LV-HIIT, HV-HIIT, and yoga was associated with improvements in binge eating behaviors, negative emotional states, and VO2max in inactive young women with binge eating, with no evidence of differential efficacy between interventions. LV-HIIT may be promising because of its shorter duration and higher adherence; however, this requires confirmation in larger trials.

## 1. Introduction

The global burden of eating disorders continues to rise [1]. Among these, Binge Eating Disorder (BED), which involves repeated loss-of-control eating episodes [2], is estimated to affect 1.5% of women and 0.3% of men globally [3]. Young women represent a high-risk population for BED [4], and the disorder occurs in both normal-weight and overweight/obese individuals [5,6].

In addition to binge eating behaviors, BED is associated with multiple psychological and physical health problems [3,7]. These include obesity-related health risks, body image disturbance, emotional dysregulation, impulsivity, reduced physical activity levels, impaired physical fitness, and elevated cardiometabolic risk [3,7,8,9]. Current first-line treatments, including cognitive behavioral therapy and pharmacotherapy, are effective in reducing binge eating behaviors; however, their effects on broader psychological and physical comorbidities remain limited, and some patients exhibit a suboptimal response or experience adverse effects [7]. These limitations highlight the need for adjunctive interventions capable of simultaneously targeting multiple domains of dysfunction.

In recent years, exercise interventions have received increasing attention as adjunctive strategies for alleviating binge eating (BE) [7,10]. Historically, exercise interventions were often considered unsuitable for individuals with eating disorders, primarily because patients with anorexia nervosa and bulimia nervosa frequently misuse exercise as a compensatory behavior to offset excessive caloric intake [11]. However, binge eating disorder is generally not accompanied by compensatory behaviors [2]. Binge eating episodes are often associated with psychological distress and may serve as maladaptive coping responses to anxiety and depression [12,13]. Research has shown that exercise can not only alleviate anxiety and depressive symptoms in individuals with binge eating, improve emotional regulation [7,14,15,16], and enhance body image perception [17,18] through psychological pathways, but also exert beneficial effects by modulating appetite-related mechanisms and food reward responses [19]. In addition, exercise contributes to improvements in cardiorespiratory fitness, metabolic health, and overall physical health [20].

Yoga has been preliminarily recognized as an adjunctive intervention strategy for eating disorders [21], and our previous study demonstrated greater improvements in binge eating symptoms following yoga compared with usual care [10]. It served as an active comparator, allowing a direct comparison between HIIT and a commonly used mind–body exercise intervention for the management of binge eating behaviors. As a low-intensity mind–body exercise, yoga may reduce binge eating behaviors by enhancing emotional awareness, alleviating negative emotions, and improving body image perception [10,22]. However, findings regarding the effects of yoga on physical fitness remain inconsistent [23,24,25,26,27,28].

High-intensity interval training (HIIT), as a time-efficient and effective form of exercise, has gained increasing attention in recent years [29,30], and a large body of evidence has demonstrated its effectiveness in improving cardiorespiratory fitness [31] and metabolic health [32,33]. Prior research indicates that HIIT can promote spontaneous and unconscious regulation of dietary choices in young individuals, thereby facilitating healthier eating patterns [34]. In addition, some studies suggest that HIIT may influence appetite-related hormone levels [35,36]; however, findings remain inconsistent across studies [37]. Meanwhile, HIIT has been shown to modulate the kynurenine pathway, shifting its metabolism from neurotoxic branches toward more neuroprotective pathways, a change associated with reduced impulsivity [38], which may be relevant to impulse-control aspects of eating behavior [39].

However, research examining the use of HIIT among individuals with binge eating (BE) remains limited. To date, only one study has examined the effects of HIIT among individuals exhibiting BE and reported no significant improvements in binge eating behaviors [10]. Notably, this study did not distinguish between different HIIT training volumes. Given that low-volume high-intensity interval training (LV-HIIT) (≤15 min) [33] and high-volume high-intensity interval training (HV-HIIT) (25–40 min) differ in total exercise volume, perceived exertion and adherence [40], their effects on binge eating behaviors may be different. Therefore, the present study is the first to differentiate HIIT into LV-HIIT and HV-HIIT and compare their effects with those of yoga in individuals with BE.

## 2. Materials and Methods

### 2.1. Participants

A total of 137 female college students were recruited through campus posters. Following screening with the Binge Eating Scale (BES), a final sample of 55 participants was enrolled in the study.

Inclusion criteria: (1) females aged 18–30 years; (2) body mass index (BMI) ranging from 18.5 to 28 kg/m^2^; (3) physically inactive, defined as engaging in no regular exercise for at least 3 months, specifically fewer than three sessions per week of planned moderate-intensity physical activity lasting ≥ 30 min per session; (4) a normal menstrual cycle; and (5) BES ≥ 18, with no history of psychiatric disorders or prior psychological treatment.

Exclusion criteria: (1) pregnancy or planned pregnancy during the study; (2) competitive athletes; (3) severe psychiatric disorders; (4) receipt of cognitive behavioral therapy (CBT) within the past 2 years, or a diagnosis of anorexia nervosa, bulimia nervosa, or other specified eating disorders; (5) dyslipidemia, orthopedic disorders, history of stroke, use of medications affecting exercise performance, or other conditions limiting physical activity; and (6) cardiovascular, metabolic, or renal diseases, or related clinical symptoms.

Sample size was estimated using G*Power 3.1 for a two-way repeated-measures ANOVA (within–between interaction). With a medium effect size (f = 0.25), α = 0.05, power = 0.80, 3 groups, and 2 measurement points, the required total sample size was 42. To account for ~20% attrition, 55 participants were recruited, exceeding the minimum requirement.

Ethical approval for this study was obtained from the Ethics Committee of the Exercise Science Laboratory at Beijing Sport University (BSU2024116H). Written informed consent was obtained from all participants before enrollment. In addition, the trial was registered in the Chinese Clinical Trial Registry (ChiCTR2600122300).

### 2.2. Randomization and Blinding

After completion of baseline assessments, participants were randomly assigned to the LV-HIIT (*n* = 19), HV-HIIT (*n* = 18), or yoga group (*n* = 18) using a computer-generated randomization sequence created by an independent researcher with SPSS (version 27.0). Group allocation was implemented by personnel not involved in participant recruitment, assessment, or intervention delivery. Allocation concealment was maintained throughout the enrollment process, and intervention staff were blinded to group assignments prior to participant allocation.

### 2.3. Intervention

Participants in the LV-HIIT and HV-HIIT groups completed two cycle ergometer training sessions per week (Technogym, Cesena, Italy), whereas participants in the yoga group attended two structured yoga sessions per week. All sessions were separated by at least 1 day, and the intervention lasted 8 weeks (16 sessions in total).

#### 2.3.1. LV-HIIT

The LV-HIIT protocol followed a 5 × 1 min interval model, consisting of a 3-min warm-up at 50 W, five 1-min high-intensity intervals at 85–95% maximum heart rate (HRmax) interspersed with 1-min active recovery at 76% HRmax, and a 3-min cool-down at 50 W. Each session lasted approximately 15 min. Exercise intensity progressed from 85–90% HRmax during weeks 1–4 to 90–95% HRmax during weeks 5–8 [41].

Predicted HRmax was calculated using the age-based equation proposed by Gellish et al.: HRmax = 208 − 0.7 × age [42].

#### 2.3.2. HV-HIIT

The HV-HIIT protocol followed a classic 4 × 4 min interval model, consisting of a 3-min warm-up at 50 W, four 4-min high-intensity intervals at 85–95% HRmax interspersed with 3-min active recovery at 70–75% HRmax, and a 3-min cool-down at 50 W. Each session lasted approximately 30–35 min. Exercise intensity progressed from 85–90% HRmax during weeks 1–4 to 90–95% HRmax during weeks 5–8 [41].

Heart rate (HR) was continuously monitored during each HIIT session to ensure that participants reached the prescribed target intensity zone.

#### 2.3.3. Yoga

The yoga intervention consisted of 5 min of breathing exercises, 45 min of Hatha yoga practice, and 10 min of deep relaxation. The instructor had completed at least 200 h of certified yoga training and received additional training from the research team. All sessions were conducted at the Sports Rehabilitation Medical Center. The 45-min asana practice was composed of sequences selected from a standardized pool of postures (e.g., standing, balancing, seated, kneeling, prone, supine, and transitional poses), with the instructor selecting postures according to the principles of mindfulness and breath-synchronized movement to ensure each session included a balanced representation of forward folds, backbends, twists, side bends, and hip-openers. To ensure progression, foundational postures were emphasized in the first two weeks, with more challenging variations and faster transitions introduced progressively from weeks 3 to 8. Intensity was monitored using the Borg Rating of Perceived Exertion (RPE) (6–20 scale), with participants asked to maintain an RPE of 12–14 during practice. The estimated energy expenditure for each yoga session was approximately 192 kcal. The yoga intervention protocol was developed based on previously published studies [10].

### 2.4. Outcomes

Outcome measures included: (1) binge eating behaviors assessed using the BES; (2) negative emotional states assessed using the Depression Anxiety Stress Scales-21 (DASS-21); and (3) physical fitness indicators, including body composition (body fat percentage and waist circumference) and cardiorespiratory fitness (maximal oxygen uptake, VO2max).

#### 2.4.1. BES

The BES was used to assess the severity of binge eating behaviors [43]. Total scores range from 0 to 46, with higher scores indicating greater symptom severity. A score of ≥18 was considered indicative of clinically significant binge eating behaviors. In the present study, the 16-item scale demonstrated good internal consistency (Cronbach’s α = 0.84). Additional details are presented in Table 1.

#### 2.4.2. DASS-21

The scale was used to assess symptoms of depression, anxiety, and stress [44]. Higher scores indicate greater symptom severity. In the present study, Cronbach’s α coefficients for the depression, anxiety, and stress subscales were 0.89, 0.80, and 0.85, respectively, indicating good internal consistency. Detailed scoring criteria are provided in Table 1.

#### 2.4.3. Physical Fitness Indicators

Body composition and waist circumference: Body fat percentage was assessed by bioelectrical impedance analysis with an InBody 770 device (InBody Co., Ltd., Seoul, Republic of Korea). Body composition was measured under standardized conditions, with participants instructed to avoid exercise and large meals prior to testing. Waist circumference was recorded using a non-elastic tape measure (AKTION, Zhejiang, China) at the midpoint between the lowest rib and the iliac crest following standardized anthropometric procedures [45].

Cardiorespiratory fitness: Cardiorespiratory fitness was assessed using a symptom-limited graded exercise test on a cycle ergometer. After a 1-min warm-up at 10 W, workload began at 50 W and increased by 20 W at each stage while participants maintained a cadence of 50 ± 5 rpm. Oxygen uptake was continuously measured using a portable metabolic gas analysis system (COSMED, Rome, Italy), and VO2max was defined as the highest oxygen uptake value attained during the test. Heart rate was continuously monitored using a chest-strap monitor (Polar, Kempele, Finland), and RPE was recorded during the final minute of each stage. The test was terminated upon volitional exhaustion or when predefined termination criteria were met, including RER ≥ 1.10, RPE ≥ 19, participant request, or clinical signs and symptoms indicating exercise intolerance. Peak heart rate achieved during the test was used as an estimate of HRmax [46].

### 2.5. Statistical Analysis

IBM SPSS Statistics version 27.0 was used for data analysis. Baseline variables were compared among groups using one-way ANOVA when parametric assumptions were satisfied; otherwise, equivalent nonparametric analyses were performed.

The intention-to-treat (ITT) analysis was conducted using linear mixed models (LMM) with all 55 randomized participants, utilizing all available data under MAR without imputation. The per-protocol (PP) analysis was performed as a supplement using two-way repeated-measures ANOVA on completers (*n* = 50).

Adherence rates (defined as completed vs. non-completed participation) were compared among groups using Pearson’s chi-square test. The number of training sessions attended was analyzed using one-way ANOVA. Heart rate data were reported descriptively and were not subjected to inferential statistical testing.

Mean changes were expressed as 95% confidence intervals (CIs), calculated as the mean ± 1.96 × standard errors (SEs), assuming a normal distribution.

To account for multiple testing, Benjamini–Hochberg FDR correction was applied to the Time effects in the ITT analysis (q < 0.05). Statistical significance was set at *p* < 0.05 for all other tests.

## 3. Results

### 3.1. Participant Flow and Baseline Characteristics

A total of 55 participants were randomly assigned to the LV-HIIT group (*n* = 19), HV-HIIT group (*n* = 18), and yoga group (*n* = 18). Baseline characteristics, including age, height, BMI, International Physical Activity Questionnaire (IPAQ), BES, DASS-21 and its subscales (DASS-D, DASS-A, DASS-S), waist circumference, body fat percentage, and VO2max, are presented in Table 2. No significant between-group differences were observed at baseline (all *p* > 0.05).

The intention-to-treat (ITT) analysis included all 55 randomized participants, and linear mixed models were applied to all available data under the assumption of missing at random, with no imputation for missing values. The per-protocol (PP) analysis, in contrast, included participants who completed at least 12 out of the 16 training sessions. According to this criterion, all 19 participants in the LV-HIIT group were eligible; one participant in the HV-HIIT group was excluded (*n* = 17); and four participants in the yoga group were excluded (*n* = 14). Ultimately, 50 participants (LV-HIIT group: *n* = 19; HV-HIIT group: *n* = 17; yoga group: *n* = 14) were included in the PP analysis.

No serious adverse events occurred during the intervention in any group.

**Figure 1:**
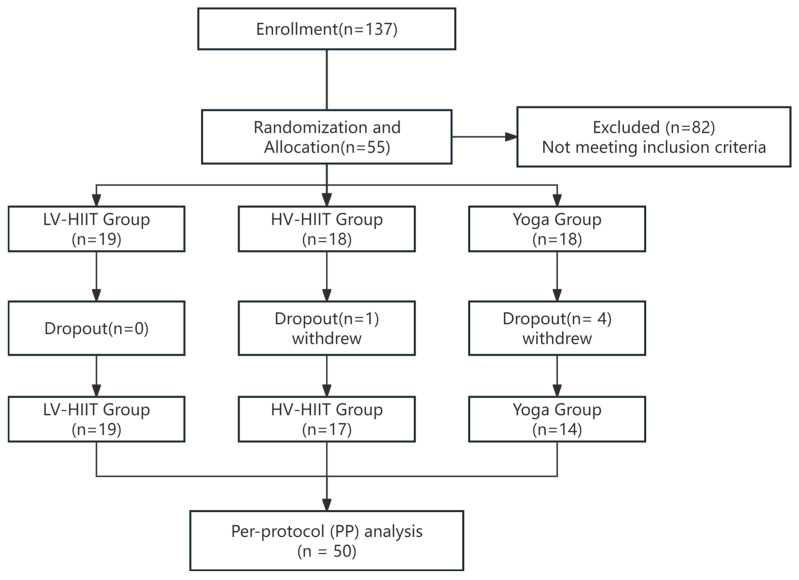
Flow chart of the experiment.

Further details are illustrated in Figure 1.

### 3.2. Binge Eating Outcomes (BES)

**Figure 2:**
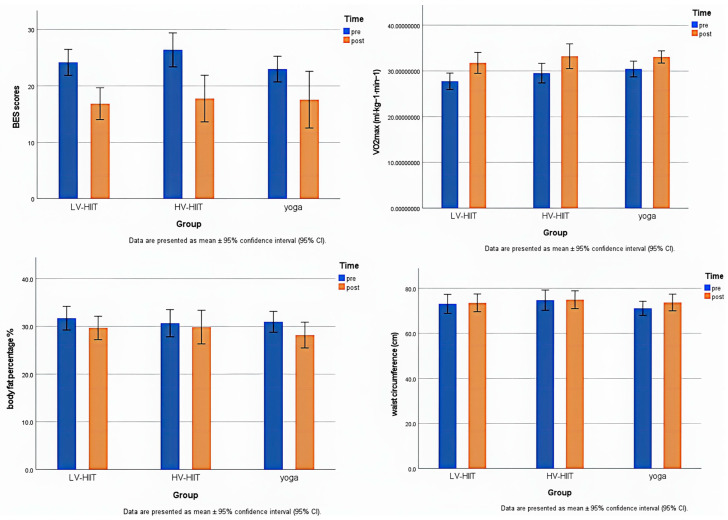
Changes in BES, VO2max, body fat percentage and waist across baseline and post-intervention among the three groups. BES, Binge Eating Scale; VO2max, maximal oxygen uptake.

The linear mixed-model results with BES as the dependent variable revealed a significant main effect of Time (F (1, 99) = 32.43, *p* < 0.001, partial η^2^ = 0.25), with binge-eating symptoms decreasing significantly across all three groups post-intervention (estimated marginal means: 24.51 vs. 17.36) (Figure 2). Neither the main effect of Group (F (2, 99) = 0.81, *p* = 0.447, partial η^2^ = 0.02) nor the Group × Time interaction effect (F (2, 99) = 0.52, *p* = 0.594, partial η^2^ = 0.01) reached statistical significance.

Supplemental description of the per-protocol (PP) analysis: Mean changes in BES were −7.32 ± 6.16 (95% CI −10.09 to −4.55) in the LV-HIIT group, −8.94 ± 7.57 (95% CI −12.55 to −5.33) in the HV-HIIT group, and −5.79 ± 8.15 (95% CI −10.06 to −1.52) in the yoga group.

### 3.3. Negative Emotional States (DASS-21)

#### 3.3.1. Total DASS-21 Score

**Figure 3:**
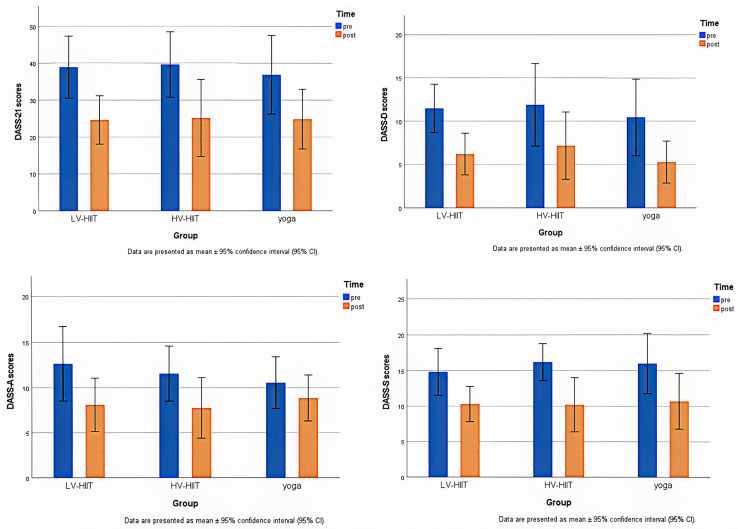
Changes in DASS-21, DASS-A, DASS-D and DASS-S scores across baseline and post-intervention among the three groups. DASS-21, Depression Anxiety Stress Scale-21; DASS-D, depression subscale of the DASS-21; DASS-A, anxiety subscale of the DASS-21; DASS-S, stress subscale of the DASS-21.

The linear mixed-model results with the DASS-21 total score as the dependent variable revealed a significant main effect of Time (F (1, 99) = 15.29, *p* < 0.001, partial η^2^ = 0.13), with overall psychological distress levels decreasing significantly across all three groups post-intervention (estimated marginal means: 38.51 vs. 24.88) (Figure 3). Neither the main effect of Group (F (2, 99) = 0.06, *p* = 0.938, partial η^2^ = 0.001) nor the Group × Time interaction effect (F (2, 99) = 0.05, *p* = 0.952, partial η^2^ = 0.001) reached statistical significance.

Supplemental description of the per-protocol (PP) analysis: Mean changes were −14.32 ± 12.76 (95% CI −20.34 to −8.30) in the LV-HIIT group, −14.24 ± 14.28 (95% CI −21.44 to −7.04) in the HV-HIIT group, and −14.86 ± 18.07 (95% CI −25.30 to −4.42) in the yoga group.

#### 3.3.2. Depression (DASS-D)

The linear mixed-model results with depression as the dependent variable revealed a significant main effect of Time (F (1, 99) = 12.89, *p* = 0.001, partial η^2^ = 0.12), with depression scores decreasing significantly across all three groups post-intervention (estimated marginal means: 11.27 vs. 6.28) (Figure 3). Neither the main effect of Group (F (2, 99) = 0.45, *p* = 0.638, partial η^2^ = 0.01) nor the Group × Time interaction effect (F (2, 99) = 0.02, *p* = 0.985, partial η^2^ < 0.001) reached statistical significance.

Supplemental description of the per-protocol (PP) analysis: Mean changes were −5.26 ± 3.90 (95% CI −7.12 to −3.40) in the LV-HIIT group, −4.71 ± 4.84 (95% CI −7.19 to −2.23) in the HV-HIIT group, and −6.29 ± 8.41 (95% CI −10.89 to −1.69) in the yoga group.

#### 3.3.3. Anxiety (DASS-A)

The linear mixed-model results with anxiety as the dependent variable revealed a significant main effect of Time (F (1, 99) = 6.95, *p* = 0.010, partial η^2^ = 0.07), with anxiety scores decreasing significantly across all three groups post-intervention (estimated marginal means: 11.60 vs. 8.20) (Figure 3). Neither the main effect of Group (F (2, 99) = 0.14, *p* = 0.872, partial η^2^ = 0.003) nor the Group × Time interaction effect (F (2, 99) = 0.43, *p* = 0.649, partial η^2^ = 0.01) reached statistical significance.

Supplemental description of the per-protocol (PP) analysis: Mean changes were −4.53 ± 5.33 (95% CI −7.08 to −1.98) in the LV-HIIT group, −3.88 ± 4.97 (95% CI −6.40 to −1.36) in the HV-HIIT group, and −2.57 ± 4.67 (95% CI −5.32 to 0.18) in the yoga group.

#### 3.3.4. Stress (DASS-S)

The linear mixed-model results with stress as the dependent variable revealed a significant main effect of Time (F (1, 99) = 15.97, *p* < 0.001, partial η^2^ = 0.14), with stress scores decreasing significantly across all three groups post-intervention (estimated marginal means: 15.67 vs. 10.40) (Figure 3). Neither the main effect of Group (F (2, 99) = 0.14, *p* = 0.871, partial η^2^ = 0.003) nor the Group × Time interaction effect (F (2, 99) = 0.11, *p* = 0.898, partial η^2^ = 0.002) reached statistical significance.

Supplemental description of the per-protocol (PP) analysis: Mean changes were −4.53 ± 5.88 (95% CI −7.18 to −1.87) in the LV-HIIT group, −5.65 ± 7.32 (95% CI −8.81 to −2.49) in the HV-HIIT group, and −6.00 ± 6.47 (95% CI −9.40 to −2.60) in the yoga group.

### 3.4. Physical Fitness Outcomes

#### 3.4.1. Body Fat Percentage

The linear mixed-model results with body fat percentage as the dependent variable revealed that neither the main effect of Time (F (1, 99) = 3.16, *p* = 0.079, partial η^2^ = 0.03) (Figure 2), nor the main effect of Group (F (2, 99) = 0.38, *p* = 0.686, partial η^2^ = 0.01), nor the Group × Time interaction effect (F (2, 99) = 0.28, *p* = 0.754, partial η^2^ = 0.01) reached statistical significance.

The per-protocol (PP) analysis further revealed a significant main effect of Time (F(1, 47) = 5.76, *p* = 0.020, partial η^2^ = 0.11), with changes in body fat percentage being −2.04 ± 1.85 (95% CI: −2.92, −1.16) for the LV-HIIT group, −1.11 ± 9.05 (95% CI: −5.55, 3.33) for the HV-HIIT group, and −2.54 ± 2.35 (95% CI: −3.93, −1.15) for the yoga group.

#### 3.4.2. Waist Circumference

The linear mixed-model results with waist circumference as the dependent variable revealed that neither the main effect of Time (F(1, 99) = 0.49, *p* = 0.486, partial η^2^ = 0.005) (Figure 2), nor the main effect of Group (F(2, 99) = 0.80, *p* = 0.452, partial η^2^ = 0.02), nor the Group × Time interaction effect (F(2, 99) = 0.23, *p* = 0.793, partial η^2^ = 0.005) reached statistical significance.

#### 3.4.3. Cardiorespiratory Fitness (VO2max)

The linear mixed-model results with VO2max as the dependent variable revealed a significant main effect of Time (F (1, 99) = 18.20, *p* < 0.001, partial η^2^ = 0.16), with cardiorespiratory fitness improving significantly across all three groups post-intervention (estimated marginal means: 29.22 vs. 32.64 mL/kg/min) (Figure 2). Neither the main effect of Group (F (2, 99) = 2.41, *p* = 0.095, partial η^2^ = 0.05) nor the Group × Time interaction effect (F (2, 99) = 0.26, *p* = 0.770, partial η^2^ = 0.005) reached statistical significance.

Supplemental description of the per-protocol (PP) analysis: Mean changes were 4.03 ± 3.54 (95% CI 2.44 to 5.62) in the LV-HIIT group, 3.84 ± 3.49 (95% CI 2.18 to 5.50) in the HV-HIIT group, and 3.00 ± 3.84 (95% CI 0.99 to 5.01) in the yoga group.

After Benjamini–Hochberg correction for multiple comparisons, all significant findings from the ITT analysis (BES, DASS-21 total score and subscales, and VO2max) remained significant (all q < 0.05), whereas body fat percentage (q = 0.120) and waist circumference (q = 0.486) remained non-significant. Notably, however, body fat percentage showed a significant reduction in the PP analysis (*p* = 0.020), a discrepancy addressed in the Discussion.

Detailed results are presented in Table 3. For the results of the per-protocol (PP) analysis, please refer to Appendix A Table A1.

### 3.5. Training Adherence and Exercise Intensity

Adherence rates were 100% (19/19) in the LV-HIIT group, 94.4% (17/18) in the HV-HIIT group, and 77.8% (14/18) in the yoga group. Although a decreasing trend in adherence was observed across groups, the between-group difference was not statistically significant (χ^2^ = 5.93, *df* = 2, *p* = 0.052).

A one-way ANOVA revealed a significant difference in the number of completed training sessions among the three groups (F = 3.524, *p* = 0.037). Post hoc comparisons using Tukey’s test indicated that the LV-HIIT group (13.11 ± 1.76 sessions) completed significantly more training sessions than the yoga group (10.39 ± 3.27 sessions), with a mean difference of 2.72 sessions (*p* = 0.012). No significant differences were observed between the LV-HIIT and HV-HIIT groups (11.78 ± 3.95 sessions; *p* = 0.405), or between the HV-HIIT and yoga groups (*p* = 0.492). The assumption of homogeneity of variances was satisfied as assessed by Levene’s test (*p* = 0.228).

During the 8-week intervention, peak heart rates (HRmax) recorded during training in the LV-HIIT and HV-HIIT groups were 163.94 ± 11.62 bpm and 162.83 ± 7.64 bpm, respectively, corresponding to 76–96% of individual HRmax, indicating vigorous-intensity physical activity.

Detailed results are presented in Table 4.

### 3.6. Exercise Time Efficiency

LV-HIIT required approximately 15 min per session, compared with 31 min for HV-HIIT and 60 min for the yoga intervention. Consequently, LV-HIIT was the most time-efficient intervention, requiring only approximately 48% of the session duration of HV-HIIT and 25% of that of yoga.

## 4. Discussion

This study is the first to compare the effects of LV-HIIT, HV-HIIT, and yoga on binge eating behaviors, negative emotional states, and physical fitness in young female college students.

The present study found that eight weeks of LV-HIIT, HV-HIIT, and yoga all improved binge eating behaviors and negative emotional states, with no significant between-group differences.

Regarding physical fitness, all three interventions significantly improved cardiorespiratory fitness (VO2max). However, no significant changes were observed in body fat percentage or waist circumference in the ITT analysis.

### 4.1. Yoga

The study further suggests that yoga may serve as a potential adjunctive intervention for binge eating problems. This finding is broadly consistent with previous studies on binge eating or emotional eating [10,47,48].

Previous studies have demonstrated a close association between binge eating behaviors and negative emotional states [49,50]. Individuals with BE may engage in binge eating behaviors to alleviate or escape from aversive emotional states [12,13,51]. Therefore, the reduction in negative affect is considered an important target in BED interventions [52,53]. In addition, multiple studies have also identified negative emotions as a prognostic indicator of eating disorders [11,22,54]. Richards et al. found that yoga practice may help reduce emotional eating and loss-of-control binge eating behaviors, an effect that may be associated with reduced perceived stress levels [47]. The present study similarly observed that yoga improved negative emotional states, consistent with previous findings [55,56]. In addition, yoga has been suggested to exert beneficial psychological regulatory effects in a range of mental health-related conditions [57,58,59]. As an integrative mind–body exercise, yoga emphasizes body awareness, mindful experience, and nonjudgmental self-acceptance [60,61]. Previous studies have suggested that yoga may alleviate stress responses and emotional dysregulation by improving autonomic nervous system function, reducing hyperactivation of the hypothalamic–pituitary–adrenal (HPA) axis, and enhancing vagal tone [62,63]. Overall, yoga may improve binge eating behaviors through the psychological pathways described above.

The present study found that yoga did not improve body composition in young female college students with binge eating based on the ITT analysis. This finding is inconsistent with some previous studies [64,65,66]. However, it should be noted that those studies primarily targeted overweight or obese populations [64,65,66]. Existing studies and meta-analyses have shown that the beneficial effects of yoga on body composition are primarily observed in overweight or obese individuals, while the effects are limited or not significant in individuals with normal body weight [25,67]. Although the mean BMI of the present study sample was within the normal range, both normal-weight and overweight/obese participants were included, with a relatively large standard deviation indicating substantial inter-individual variability. This sample composition may limit direct comparability with previous studies that primarily focused on populations with a single body weight characteristic.

However, previous studies have confirmed an association between binge eating disorder and obesity [68,69], and more than 65% of individuals with BED are obese [70]. Obesity has been associated with weight-related psychological distress [66,71,72], which may negatively affect both physical and mental health [73]. Therefore, future studies should conduct stratified analyses across different weight categories and further explore the relationship between binge eating behaviors and body composition. In addition, eating behaviors and energy intake should be monitored to determine whether these factors also influence body composition in individuals with binge eating behaviors.

Regarding cardiorespiratory fitness, the present study found that yoga can improve VO2max; however, findings from previous studies remain inconsistent [24,74]. This discrepancy may be primarily attributed to heterogeneity in intervention protocols, exercise intensity, and study populations [75]. Due to the diversity of yoga styles, considerable variations exist among studies regarding the forms of yoga and training intensities employed [75]. The present study implemented moderate-intensity yoga, whereas several studies that failed to observe positive effects of yoga on cardiorespiratory endurance primarily utilized low-intensity yoga interventions [27,74].

### 4.2. LV-HIIT and HV-HIIT

Although LV-HIIT consisted of substantially lower training volume than HV-HIIT (5 × 1 vs. 4 × 4 structure), both protocols elicited comparable improvements in binge eating behaviors and negative emotional states. Previous studies, such as those conducted by Blundell and colleagues, have suggested that higher exercise volumes may induce compensatory increases in food intake; therefore, HV-HIIT may theoretically be more likely to increase energy intake [76]. However, this phenomenon was not observed in female college students with binge eating in the present study. These findings suggest that exercise-induced changes in eating behavior among individuals with BE should not be explained solely from the perspective of energy compensation. The beneficial effects of HIIT on binge eating behaviors may also be related to improvements in negative emotions, enhanced inhibitory control, and regulation of neuropsychological mechanisms associated with impulsive eating behaviors [38,39]. Previous studies have shown that HIIT can alleviate negative emotional states by modulating monoamine neurotransmitters, improving hypothalamic–pituitary–adrenal (HPA) axis function, and reducing inflammatory responses [77]. In addition, HIIT may enhance prefrontal cortex–related inhibitory control and attentional regulation, thereby reducing reward responsiveness to high-calorie food cues and impulsive eating tendencies [39,78].

The following studies further support this perspective: A randomized controlled trial by Martins et al. in individuals with obesity found that, under equivalent energy expenditure, HIIT did not significantly increase appetite or food reward responses compared with moderate-intensity continuous exercise [37]. In addition, although only one study has specifically investigated the effects of HIIT in individuals with binge eating behaviors [10], evidence from different populations suggests that HIIT can reduce preference for high-calorie foods and improve eating behavior regulation without increasing the risk of compensatory eating [37,39].

HIIT and yoga are two distinct types of exercise interventions that may improve binge eating behaviors through partially different underlying mechanisms. Current evidence remains limited, and existing literature suggests that HIIT may primarily reduce impulsive eating behaviors by enhancing inhibitory control [39,78], whereas yoga may act through mind–body integrative processes to improve negative emotions, enhance body awareness, and further alleviate emotional eating behaviors [21,22,48,79]. However, there is still a lack of studies directly comparing or validating the differential mechanistic pathways of these two exercise interventions in populations with binge eating behaviors.

Overall, the proposed mechanisms should be interpreted as theoretical explanations that require confirmation in future studies incorporating direct physiological and neuropsychological measurements.

Regarding cardiorespiratory fitness, HV-HIIT and LV-HIIT demonstrated similar improvements in VO2max. In HIIT training, improvements in cardiorespiratory fitness are primarily driven by exercise intensity rather than the number of repetitions or total training duration [33]. In the present study, both HIIT protocols were performed at comparable intensities, which may explain the similar outcomes observed. Furthermore, LV-HIIT, characterized by short bouts of high-intensity effort interspersed with sufficient recovery, may be analogous to “cluster set” training in resistance exercise [80]. This structure may help maintain higher peak power output during each sprint, thereby enhancing the quality of each high-intensity stimulus while optimizing movement performance. Therefore, LV-HIIT can still effectively improve cardiorespiratory fitness despite a lower total training volume [81], which may be attributable to higher-quality exercise output per bout and more appropriately structured recovery intervals [82]. Future studies may further optimize the structural design of HV-HIIT by implementing protocols characterized by shorter durations of each high-intensity bout combined with a greater number of training sets, thereby increasing overall training volume while maintaining high-quality exercise output and further clarifying the potential role of training volume in improving cardiorespiratory fitness.

Similar to the findings for yoga, the present study also found that neither LV-HIIT nor HV-HIIT improved body composition. This is also inconsistent with some previous studies [31,83,84]. Given that energy intake and dietary behaviors were not objectively assessed in the present study, it is currently difficult to determine whether the absence of changes in body composition is related to the specific characteristics of individuals with binge eating behaviors.

The present findings suggest that LV-HIIT may represent a more time-efficient and feasible exercise intervention for individuals with binge eating. Although no statistically significant differences in adherence were observed among the three groups, LV-HIIT demonstrated a significantly greater number of completed training sessions compared with yoga, suggesting potential differences in real-world implementation across exercise modalities. In addition, each LV-HIIT session required only 15 min, compared with 31 min for HV-HIIT and 60 min for yoga, indicating superior time efficiency. Globally, approximately 23.3% of adults do not meet recommended physical activity levels, with insufficient time cited as one of the main obstacles to regular exercise [85,86,87]. In this context, the short duration, high efficiency, and superior adherence associated with LV-HIIT may make it a feasible exercise intervention strategy for individuals with binge eating.

#### Limitations

This study has certain limitations. First, the relatively small sample size in the present study may have reduced statistical power, limiting the ability to detect potential differences between intervention groups. Therefore, although no significant between-group differences were observed in binge eating behaviors, negative emotional states, and cardiorespiratory fitness, these findings should not be interpreted as evidence of equivalence among the three exercise interventions. Future studies with larger sample sizes are warranted to further confirm these findings.

Second, participants were screened based on BES rather than a formal clinical diagnosis of binge eating disorder (BED), and all binge eating and psychological outcomes were assessed using self-report measures. These factors may introduce reporting bias and limit the diagnostic accuracy of participant classification. Therefore, the generalizability of the present findings to clinically diagnosed BED populations should be interpreted with caution. Future studies should incorporate structured clinical interviews and objective assessment methods to strengthen the validity of the findings.

Third, the study did not systematically monitor dietary intake, sleep quality, daily physical activity, or academic stress, all of which may have influenced the observed outcomes. Furthermore, body fat percentage was assessed using bioelectrical impedance analysis, a method that is sensitive to hydration status and measurement conditions. Although participants were assessed under standardized procedures, uncontrolled variations in fluid balance, recent food and beverage intake, and other pre-assessment factors may have affected the accuracy of body composition measurements. In addition, menstrual cycle phase was not controlled, which may have introduced further variability in body composition estimates. Future research should incorporate comprehensive monitoring of these factors.

## 5. Conclusions

An 8-week intervention of LV-HIIT, HV-HIIT, and yoga was associated with significant improvements in binge eating behaviors and negative emotional states in young female college students with binge eating. All three interventions also improved cardiorespiratory fitness, but none resulted in improvements in body composition. However, no evidence of differential effects was observed among the three exercise interventions. Given its shorter duration and higher adherence rate, LV-HIIT may represent a promising exercise option. This potential practical advantage should be confirmed in larger randomized controlled trials.

## Figures and Tables

**Table 1:** Severity levels of the assessed scales.

Subscale	Normal	Mild	Moderate	Severe	Extremely Severe
BES	0–17	18–26		≥28	/
DASS-D	0–9	10–13	14–20	21–27	≥28
DASS-A	0–7	8–9	10–14	15–19	≥20
DASS-S	0–14	15–18	19–25	26–33	≥34

Abbreviations: BES, Binge Eating Scale; DASS-D, depression subscale of the DASS-21; DASS-A, anxiety subscale of the DASS-21; DASS-S, stress subscale of the DASS-21.

**Table 2:** Baseline characteristics of participants before intervention (*n* = 55).

Characteristics	LV-HIIT (*n* = 19)	HV-HIIT (*n* = 18)	Yoga (*n* = 18)	*p*-Value
Age (years)	21.53 ± 2.04	21.89 ± 2.867	21.22 ± 2.211	0.706
Height (cm)	163.11 ± 4.14	167.08 ± 7.86	163.12 ± 4.82	0.068
BMI (kg/m^2^)	22.32 ± 2.75	23.36 ± 2.57	21.99 ± 2.80	0.292
IPAQ (MET min/w)	151.48 ± 6.24	150.78 ± 25.26	152.18 ± 7.49	0.159
BES	24.16 ± 4.776	26.39 ± 6.040	23.00 ± 4.589	0.145
DASS-21	38.95 ± 17.440	39.67 ± 17.839	36.89 ± 21.401	0.901
DASS-D	11.47 ± 5.767	11.89 ± 9.566	10.44 ± 8.853	0.862
DASS-A	12.63 ± 8.513	11.56 ± 6.119	10.56 ± 5.731	0.663
DASS-S	14.84 ± 6.809	16.22 ± 5.174	16.00 ± 8.430	0.809
BF%	31.70 ± 5.138	30.66 ± 5.727	30.94 ± 4.398	0.813
WC (CM)	73.11 ± 8.733	74.74 ± 9.034	71.13 ± 6.300	0.417
VO2max (mL·kg^−1^·min^−1^)	27.74 ± 3.72	29.54 ± 4.28	30.46 ± 3.447	0.09

Note: Data are presented as the mean ± SD. LV-HIIT, low-volume high-intensity interval training group; HV-HIIT, high-volume high-intensity interval training group; IPAQ, International Physical Activity Questionnaire; BMI, body mass index; BES, Binge Eating Scale; DASS-21, Depression Anxiety Stress Scale-21; DASS-D, depression subscale of the DASS-21; DASS-A, anxiety subscale of the DASS-21; DASS-S, stress subscale of the DASS-21; WC, waist circumference; BF, body fat percentage; VO2max, maximal oxygen uptake.

**Table 3:** Intention-to-treat analysis of outcomes across time points (*n* = 55).

Outcomes	Group	*n*	Pre	Post	Group *p*-Value	Time *p*-Value	BH-Adjusted q-Value	Interaction *p*-Value
		Pre/Post	M (95% CI)	M (95% CI)				
BES	LV-HIIT	19/19	24.16 (21.26, 27.06)	16.84 (13.94, 19.74)	0.447	<0.001	<0.001	0.594
	HV-HIIT	18/17	26.39 (23.41, 29.37)	17.77 (14.70, 20.83)				
	Yoga	18/14	23.00 (20.02, 25.98)	17.57 (14.19, 20.95)				
DASS-21	LV-HIIT	19/19	38.95 (30.87, 47.02)	24.63 (16.56, 32.71)	0.938	<0.001	<0.001	0.952
	HV-HIIT	18/17	39.67 (31.37, 47.96)	25.18 (16.64, 33.71)				
	Yoga	18/14	36.89 (28.59, 45.19)	24.86 (15.45, 34.26)				
DASS-D	LV-HIIT	19/19	11.47 (8.21, 14.73)	6.21 (2.95, 9.47)	0.638	0.001	0.003	0.985
	HV-HIIT	18/17	11.89 (8.54, 15.24)	7.18 (3.73, 10.62)				
	Yoga	18/14	10.44 (7.10, 13.79)	5.29 (1.49, 9.08)				
DASS-A	LV-HIIT	19/19	12.63 (9.69, 15.57)	8.11 (5.17, 11.04)	0.872	0.01	0.017	0.649
	HV-HIIT	18/17	11.56 (8.54, 14.57)	7.77 (4.66, 10.87)				
	Yoga	18/14	10.56 (7.54, 13.57)	8.86 (5.44, 12.28)				
DASS-S	LV-HIIT	19/19	14.84 (11.79, 17.90)	10.32 (7.26, 13.37)	0.871	<0.001	<0.001	0.898
	HV-HIIT	18/17	16.22 (13.08, 19.36)	10.24 (7.00, 13.47)				
	Yoga	18/14	16.00 (12.86, 19.14)	10.71 (7.15, 14.28)				
WC (cm)	LV-HIIT	19/19	73.11 (69.52, 76.69)	73.55 (69.97, 77.13)	0.452	0.486	0.486	0.793
	HV-HIIT	18/17	74.74 (71.06, 78.42)	74.94 (71.15, 78.72)				
	Yoga	18/14	71.13 (67.45, 74.81)	73.74 (69.56, 77.91)				
BF (%)	LV-HIIT	19/19	31.70 (29.25, 34.15)	29.66 (27.21, 32.11)	0.686	0.079	0.120	0.754
	HV-HIIT	18/17	30.66 (28.14, 33.17)	29.84 (27.25, 32.43)				
	Yoga	18/14	30.94 (28.42, 33.45)	28.17 (25.32, 31.02)				
VO2max (mL·kg^−1^·min^−1^)	LV-HIIT	19/19	27.74 (25.87, 29.62)	31.77 (29.90, 33.65)	0.095	<0.001	<0.001	0.770
	HV-HIIT	18/17	29.54 (27.61, 31.47)	33.23 (31.25, 35.22)				
	Yoga	18/14	30.46 (28.53, 32.39)	33.09 (30.91, 35.28)				

Note: Data are presented as the mean ± SD. LV-HIIT, low-volume high-intensity interval training group; HV-HIIT, high-volume high-intensity interval training group; BES, Binge Eating Scale; DASS-21, Depression Anxiety Stress Scale-21; DASS-D, depression subscale of the DASS-21; DASS-A, anxiety subscale of the DASS-21; DASS-S, stress subscale of the DASS-21; WC, waist circumference; BF, body fat percentage; VO2max, maximal oxygen uptake. Benjamini–Hochberg false discovery rate correction was applied across the eight outcome-specific time effects.

**Table 4:** Subject training data, compliance, and acceptance status.

Variable	LV-HIIT (*n* = 19)	HV-HIIT (*n* = 18)	Yoga (*n* = 18)	Statistics
Total number of completions	13.11 ± 1.76	11.78 ± 3.95	10.39 ± 3.27	F = 3.524, *p* = 0.037
Adherence rate (%)	100	94.4	77.8	χ^2^ = 5.93, *df* = 2, *p* = 0.052
Maximum heart rate during exercise (HRmax)	163.94 ± 11.62	162.83 ± 7.64	/	
Total training time (min)	15	31	60	

## Data Availability

The datasets generated and/or analyzed during the current study are available from the corresponding authors upon reasonable request. The data are not publicly available due to privacy.

## Appendix A

**Table A1:** Pre- and post-intervention outcomes based on per-protocol analysis (*n* = 50).

Outcomes	Group	Pre	Post	ΔPre–Post	Group *p*-Value	Time *p*-Value	BH-Adjusted q-Value	Interaction *p*-Value
		M ± SD	M ± SD	M ± SD				
BES	LV-HIIT	24.16 ± 4.78	16.84 ± 5.84	−7.32 ± 6.16	0.553	<0.001	<0.001	0.486
	HV-HIIT	26.71 ± 6.07	17.76 ± 8.04	−8.94 ± 7.57				
	Yoga	23.36 ± 4.72	17.57 ± 8.71	−5.79 ± 8.15				
DASS-21	LV-HIIT	38.95 ± 17.44	24.63 ± 13.60	−14.32 ± 12.76	0.994	<0.001	<0.001	0.992
	HV-HIIT	39.41 ± 18.35	25.18 ± 20.31	−14.24 ± 14.28				
	Yoga	39.71 ± 23.08	24.86 ± 14.00	−14.86 ± 18.07				
DASS-D	LV-HIIT	11.47 ± 5.77	6.21 ± 4.98	−5.26 ± 3.90	0.896	<0.001	<0.001	0.748
	HV-HIIT	11.88 ± 9.86	7.18 ± 7.55	−4.71 ± 4.84				
	Yoga	11.57 ± 9.58	5.29 ± 4.20	−6.29 ± 8.41				
DASS-A	LV-HIIT	12.63 ± 8.51	8.11 ± 6.16	−4.53 ± 5.33	0.945	<0.001	<0.001	0.616
	HV-HIIT	11.65 ± 6.29	7.76 ± 6.55	−3.88 ± 4.97				
	Yoga	11.43 ± 5.46	8.86 ± 4.42	−2.57 ± 4.67				
DASS-S	LV-HIIT	14.84 ± 6.81	10.32 ± 5.17	−4.53 ± 5.88	0.865	<0.001	<0.001	0.791
	HV-HIIT	15.88 ± 5.12	10.24 ± 7.44	−5.65 ± 7.32				
	Yoga	16.71 ± 9.37	10.71 ± 6.77	−6.00 ± 6.47				
WC (cm)	LV-HIIT	73.11 ± 8.73	73.55 ± 8.20	/	0.554	0.497	0.497	0.569
	HV-HIIT	75.49 ± 8.72	74.94 ± 7.70	/				
	Yoga	71.46 ± 6.59	73.74 ± 6.43	/				
BF (%)	LV-HIIT	31.70 ± 5.14	29.66 ± 5.09	−2.04 ± 1.85	0.746	0.02	0.023	0.762
	HV-HIIT	30.95 ± 5.76	29.84 ± 6.85	−1.11 ± 9.05				
	Yoga	30.71 ± 4.67	28.17 ± 4.68	−2.54 ± 2.35				
VO2max (mL·kg^−1^·min^−1^)	LV-HIIT	27.74 ± 3.72	31.77 ± 4.76	4.03 ± 3.54	0.255	<0.001	<0.001	0.394
	HV-HIIT	29.40 ± 4.37	33.23 ± 5.26	3.84 ± 3.49				
	Yoga	30.66 ± 3.77	33.09 ± 2.31	3.00 ± 3.84				

Note: Data are presented as the mean ± SD. LV-HIIT, low-volume high-intensity interval training group; HV-HIIT, high-volume high-intensity interval training group; BES, Binge Eating Scale; DASS-21, Depression Anxiety Stress Scale-21; DASS-D, depression subscale of the DASS-21; DASS-A, anxiety subscale of the DASS-21; DASS-S, stress subscale of the DASS-21; WC, waist circumference; BF, body fat percentage; VO2max, maximal oxygen uptake. Data were analyzed using a per-protocol (PP) approach including participants who completed the intervention. Benjamini–Hochberg false discovery rate correction was applied across the eight outcome-specific time effects.
